# Surgery

**Published:** 1881-02

**Authors:** 


### SURGERY.

SURGICAL ANALGESIA. Dr. Bossis. (*Le Praticien*, Sept. 20, 1880. Page 454.)

Under the name of surgical analgesia Dr. Bossis describes insensibility to pain, accompanied by persistence and followed by the integrity of the intellectual faculties and consciousness; it is easily obtained by injecting 15 milligrams of morphine and inhaling twenty minutes after a very little choloroform. Here are the conclusions of M. Bossis:

1. There can be obtained in man, with a little attention, by the combined action of chloroform and morphine, a state of complete insensibility to pain, with the conservation at least partial of his intelligence, tactile, auditive and visual sensibility, and voluntary movements. We propose to call this state *surgical analgesia.*

2. In a practical point of view the analgesia obtained by the combined action differs completely from the semi-anæsthesia obtained by chloroform or ether employed alone, in that it is not preceded nor accompanied by a period of hyperæsthesia, with violent excitation and tendency to the exaggeration of reflex action of the heart, and, in consequence, syncope. The anæsthesia is quite comparable to the period of tolerance of chloroform employed alone.

3. This procedure, joined to the benefits of the combined action, in the initial period, has the great advantage of avoiding the dangers which accompany or follow complete anæsthesia.

4. Thus far the employment of analgesia has proven quite free from danger. However, before the question is definitely settled, new researches are needed; awaiting the advantages which it promises this study merits the attention of surgeons.

SURGICAL TREATMENT OF HYPERTROPHIED PROSTATE. (*Le Praticien*, Sept. 20, 1880. Page 453.)

M. Lefevre, of Louvain, uses in this affection so rebellious to treatment, a compressor with two branches. The one is introduced like a catheter by the urethra; the other enters the rectum; the two branches are articulated after the manner of forceps, and allow one to subject the largest portion of the gland to the effect of an intense compression. M. Lefevre also uses a catheter provided with a reservoir of oil, which on account of this abundant lubrication on the spot, allows the instrument to readily pass into the bladder.

